# A New Remedy for Catarrh

**Published:** 1870-11

**Authors:** 


### A NEW REMEDY FOR CATARRH.

A recent number of the *Heilbronn Memorabilien*, (Germany,) contains the following resume of an article on the nature and treatment of Catarrh, by Dr. M. Frank, of Munich.

Nasal Catarrh should always be regarded as an infectious disease. The infectious principle is chiefly transmitted to the nasal passages in the air of respiration, which very soon manifests its action in a profuse watery secretion from the mucous surfaces. The affection is then propagated along the lachrymal ducts to the conjunctival membrane, into the pharynx, Eustachian tubes, larynx, trachea and bronchial tubes, with the accompanying train of symptoms in these organs; or the affection may extend down the œsophagus, into the stomach and intestinal tract, with the corresponding morbid phenomena. In most instances, where the intensity is not great, it runs its course in from seven to eleven days. With many the febrile state, sense of weakness, and feeling of discomfort generally are so great that the patient will take to his bed, or the necessity of continual cleansing of the nostrils, or sometimes the just appreciation of the danger to others, will cause him, for the time being, to avoid all society.

The constant neglect of colds, or ordinary catarrh, on account of its being regarded a trivial affection, is a matter of daily observation, and which led Hufeland to assert that a greater number of people died of it than from the pest.

Dr. Frank recommends the following treatment, which he has practiced for two years, with constantly favorable results:

Immediately on the approach of the first symptoms in the nasal passages the patient is directed to use a weak solution of the hypermanganate of soda as a disinfectant.

Enough of the hypermanganate is added to a goblet full of water to give it a cherry red color.

A handful of this solution is snuffed up the nostrils every couple of hours, using the precaution to blow out carefully after each operation. If the pharynx has become affected, the same should also be used as a gargle. Usually before the end of the second day all symptoms have disappeared. E. O. F. R.

